# Cell-to-cell diversity in protein levels of a gene driven by a tetracycline inducible promoter

**DOI:** 10.1186/1471-2199-12-21

**Published:** 2011-05-14

**Authors:** Olli-Pekka Smolander, Meenakshisundaram Kandhavelu, Henrik Mannerström, Eero Lihavainen, Shanmugapriya Kalaichelvan, Shannon Healy, Olli Yli-Harja, Matti Karp, Andre S Ribeiro

**Affiliations:** 1 Department of Signal Processing, Tampere University of Technology, P.O. Box 553, FIN - 33101 Tampere, Finland; 2 Department of Chemistry and Bioengineering, Tampere University of Technology, P. O. Box 541, FIN 33101 Tampere, Finland; 3 Manitoba Institute of Cell Biology, Winnipeg, MB, Canada; 4 Institute for Systems Biology, Seattle, WA, USA; 5 Biosensors Competence Centre, Tampere, Finland

## Abstract

**Background:**

Gene expression in *Escherichia coli *is regulated by several mechanisms. We measured in single cells the expression level of a single copy gene coding for green fluorescent protein (GFP), integrated into the genome and driven by a tetracycline inducible promoter, for varying induction strengths. Also, we measured the transcriptional activity of a tetracycline inducible promoter controlling the transcription of a RNA with 96 binding sites for MS2-GFP.

**Results:**

The distribution of GFP levels in single cells is found to change significantly as induction reaches high levels, causing the Fano factor of the cells' protein levels to increase with mean level, beyond what would be expected from a Poisson-like process of RNA transcription. In agreement, the Fano factor of the cells' number of RNA molecules target for MS2-GFP follows a similar trend. The results provide evidence that the dynamics of the promoter complex formation, namely, the variability in its duration from one transcription event to the next, explains the change in the distribution of expression levels in the cell population with induction strength.

**Conclusions:**

The results suggest that the open complex formation of the tetracycline inducible promoter, in the regime of strong induction, affects significantly the dynamics of RNA production due to the variability of its duration from one event to the next.

## Background

Stochasticity is inherent in gene expression and affects organisms' phenotypes [1,2]. For example, it is a source of cell-to-cell phenotypic diversity in monoclonal cell populations, a key feature for bacterial adaptability to fluctuating environmental conditions [3,4].

In prokaryotes, transcription starts with the binding of an RNA polymerase (RNAp) to a promoter and the formation of the closed and then the open complex [5,6]. Also, transcription and translation are dynamically coupled, since the latter starts before the former is completed and, from one transcript, several proteins can be produced. Due to this, the fluctuations in RNA levels, which are to some extent sequence dependent, propagate to protein levels [7,8]. Since noise in gene expression can be selectively advantageous [9], it is likely that several of the regulatory mechanisms of mean gene expression levels may also regulate the noise strength in RNA and protein levels. If so, they also regulate, to some extent, cell-to-cell phenotypic variability.

Gene expression is under tight regulation by multiple mechanisms [9] that act at various stages, such as transcription initiation and elongation, translation initiation and elongation [5,10-12] and post-translation modifications, such as reversible phosphorylation [13,14]. Measurements suggest that the process of formation of the open complex varies widely in duration, from a few seconds to several minutes, between different promoters and different conditions [11,15]. Comparison between natural promoters and mutated ones showed that the mean duration of this process is also sequence dependent [11,16]. So far, only mean duration times for this process have been assessed. Little is known about its variability. Also, since these measurements were conducted *in vitro*, no studies have yet determined if the variability in duration from one event to the next affects the degree of fluctuations in RNA and protein levels *in vivo*.

The studies of this process (see e.g. [5,6,11,12,15,16]) have already confirmed that the mean duration depends on a variety of factors such as temperature, concentration of magnesium and induction strength. It is thus possible that the variability may also be condition-dependent.

A recent theoretical study characterized the effects of the mean duration of this process on RNA temporal levels. It was shown that, assuming a constant duration from one event to the next, this step acts as a noise filter. However, if the distribution of durations is wide, it can result in noise amplification [17]. Another study showed that the mean duration of the promoter open complex formation can have significant effects on the dynamics of small model genetic circuits such as the 2-gene toggle switch [18].

These results are yet to be confirmed experimentally. One difficulty in doing so is the need to observe the dynamics of gene expression at the single event level. Further, these effects are likely to be observable only in a regime of strong expression, more precisely, when the expected time between transcription events is of similar order of magnitude as that of the duration of the open complex formation [17]. Since, so far, most studies of gene expression at the single event level [7,19] were made in conditions of weak expression (so as to facilitate the visualization of each molecule expressed), it is not expected that this step in transcription played any tangible effect on the dynamics of production of RNA or proteins. In the regime of strong expression, it is expected that the distribution of durations of these events becomes one of the regulators of both mean and variability in RNA temporal levels [17].

Since the mean and variability of the duration of the promoter open complex are likely to affect the dynamics of RNA production and thus the noise in RNA levels, and since protein levels likely follow RNA levels in prokaryotes [9], then the dynamics of this process also affects the degree of cell-to-cell diversity in RNA, and, consequently, protein numbers. If so, indirect assessment of the effects of promoter open complex formation may be possible from measurements of cell-to-cell variability in RNA and/or protein numbers.

In this study, from measurements of fluorescence intensity at the single cell level of GFP expressed from a gene driven by the tetracycline inducible promoter P_LtetO-1 _that we integrated into the genome, we first characterize mean and cell-to-cell variability in protein levels as a function of induction strength. We then compare the results with those from a delayed stochastic model of gene expression with parameter values extracted from measurements. Finally, we measure directly, in individual cells, the transcriptional activity of a tetracycline inducible promoter, P_tet_, controlling the expression of an RNA target for MS2-GFP [19,20]. From this, we characterize the mean and variability in the numbers of the RNA expressed by the promoter as a function of the induction strength. From all of the above, we infer the effects of the promoter open complex formation on the dynamics of gene expression and the observed cell-to-cell diversity in protein numbers in our measurements.

## Methods

### Bacterial strains and plasmids for measurements of protein levels

We engineered a new bacterial strain for this study using the λRED recombination system [21]. An intermediate lifetime green fluorescent protein, GFP(AAV) [22] (generously donated by M. Elowitz, Caltech), was placed under the control of the P_LtetO-1 _promoter [12,23] and was inserted into the *E. coli *genome at the *galK *locus using homologous recombination.

The following primers were used to create the P_LtetO-1_-GFP insert (homologous sequence underlined):

Forward:

5'TTCATATTGTTCAGCGACAGCTTGCTGTACGGCAGGCACCAGCTCTTCCGCCAGATGGAGTTCTGAGGTC3'

Reverse:

5'GTTTGCGCGCAGTCAGCGATATCCATTTTCGCGAATCCGGAGTGTAAGAATGCCTCTAGCACGCGTACC

The proper insertion was confirmed by colony-PCR using forward primer (5'GGCAGGCACCAGCTCTTC3') annealing to genomic DNA near *galK *and reverse primer (5'CACGTACTCGGATGGAAGC3') annealing to insert DNA in Kan gene.

To control and vary the expression level of the inserted gene we transformed a plasmid vector containing constitutively expressed *tetR *gene from Tn10 [10] (generously donated by M. Karp, TUT, Finland) into GFP-expressing cells [24]. The gene *tetR *codes for a repressor protein which binds at the P_LtetO-1 _promoter and represses GFP expression.

As there are several copies of the plasmid versus the single copy of promoter, TetR is available in excess in the cells. The inserted plasmid was created by removing the *lux*-genes and the *tetA *promoter from pTetLux1 [25] plasmid by PCR mediated deletion [26], followed by ligation using the following primers containing EcoRI restriction site (underlined):

Forward: 5'GGGGGAATTCGAGCTCGGTACCCG3'

Reverse: 5'GGGGGAATTC TGTCGGGTCATGTGAGCAAA3'

### Chemicals for measurements

Anhydrotetracycline, kanamycin, glycerol, and agarose used for gel-electrophoresis and microscopy were purchased from Sigma-Aldrich. Sybr-Safe from Invitrogen was used as DNA gel dye. All live-cell measurements and cultivations were performed in Luria's broth (LB) [27]. All plasmid- and PCR-purifications were done using corresponding kits from Fermentas and following the manufacturer's instructions. All PCR reagents and enzymes were purchased from Finnzymes. Phusion high-fidelity polymerase from Finnzymes was used for PCR.

### Construction of the RNA target for MS2-GFP

To detect the RNA molecules, a promoterless version of BAC clone was created by restricting out the P_lar _promoter using BamHI restriction site from the original clone (P_lar-mRFP1-96 _binding site) [19] (kind gift from Ido Golding, University of Illinois, IL). To insert the tetracycline inducible promoter, the promoter region was amplified from the pTetLux1 [25] plasmid with following primers containing BamHI restriction site (underlined):

Forward: 5'GGGATCCCTCACATGACCCGACAC 3'

Reverse: 5'GGGATCCACTGCAATCGCGATAGC 3'

The amplified product was digested with BamHI and then ligated between the BamHI digested regions of BAC clone. The ligated product was cloned into *E. coli *strain DHα-PRO. A resulting positive clone was confirmed by sequencing, followed by BLAST. The result is the F- based single copy plasmid vector P_tet-mRFP1-MS2-96bs_. To see the target RNA, a reporter plasmid was introduced to the same strain. The details of this report vector are: PZS12MS2-GFP (SC101 origin, 6-8 copies per cell, Amp^R^, P_LlaO-1 _promoter) [20] (kind gift from Philippe Cluzel, University of Chicago, IL).

### Microscopy and measurements of mRNA molecules

Cells were grown in Miller LB medium, supplemented by antibiotics. For full induction of gene expression, cells were grown overnight at 37 C with shaking (250 RPM), diluted into fresh medium to reach a final optical density of OD600 ≈ 0.3-0.5. The cells were incubated with the inducer IPTG (1 mM) for 60 min to attain full induction of MS2-GFP, so as to produce detectable amounts of protein tag for RNA. Various concentration of aTc (0, 0.1, 0.5, 1, 2 ng/ml) were used to induce the promoter expressing the target RNA. Finally, the cells were incubated at 37 C with shaking (250 RPM) for 60 min. After induction, a few microliters of culture were taken and placed between a cover-slip and a thin slab of LB/1% agarose and imaged immediately. Multiple images of cell populations were taken from each slide. Microscopy was performed at room temperature using a Nikon TE-2000U microscope equipped with a C1 confocal imaging system and a 100× magnification (1.49 NA) objective. Images were acquired with the EZ-C1 software using medium pinhole, gain 130, and 1.68 μs pixel dwell. GFP fluorescence was measured using a 488 nm argon ion laser and a 515/30 nm detection filter.

### Microscopy and measurements of protein levels

**Figure 1:**
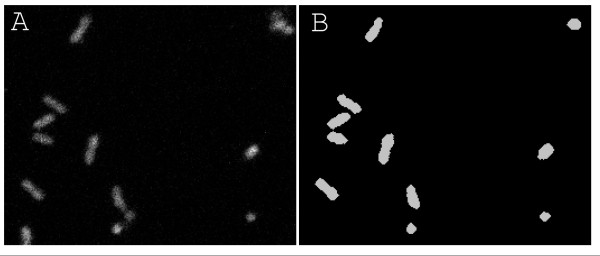
**Confocal image and a corresponding mask**. Example of a confocal image (A) used to calculate fluorescence values and a corresponding mask (B) for detecting cells.

Cells were grown overnight in LB media plus antibiotics at 37 C and with shaking (250 RPM), and then diluted with LB media to OD_600 _0.2. To induce gene expression, anhydrotetracycline was added to the diluted cell culture which was then incubated for 60 min at 37 C with shaking (250 RPM). A few microliters of induced cells were plated on a thin LB/1% agarose slab on a microscope slide, covered with #1 cover slip and immediately imaged (Figure 1).

Single cell measurements of GFP were conducted using the same microscopy setup that was used to measure mRNA molecules. Images were acquired using large pinhole, gain 120 and 1.68 μs pixel dwell. GFP fluorescence was measured using a 488 nm argon ion laser and a 515/30 nm detection filter.

### Image processing

Cells were segmented from each z-stack in a semi-automatic fashion. Each slice in the z-stack was first median filtered using a 2 by 2 window to remove noise spikes, after which an image I_SUM _was created as the sum projection of the filtered slices. A rough initial segmentation was performed by thresholding I_SUM _with a small value to obtain a mask S (Figure 1) to separate background and cells. Morphological opening using a disk-element (radius 2) was applied to S to remove objects considered as noise.

In some images, cells formed clusters, which the initial segmentation did not separate correctly. The MATLAB^® ^(2010b, The Mathworks, Natic, MA) function *regionprops *was used to compute the solidity and eccentricity for each object in S, to detect these clusters. An object was considered a cluster of cells if its solidity was smaller than 0.9 (indicating a non-convex object) or if its eccentricity was smaller than 0.7, characteristic of an object that is not as elongated as a rod-shaped bacterium. Individual cells were segmented from each detected cluster. Noting that cells' centers were brighter than their borders in I_SUM_, this was done by finding local maxima from the part of I_SUM _containing the cluster, using the extended-maxima transform [28]. Finally, objects touching the image borders as well as objects having very small or very large area were considered noise and removed from S. Finally, poorly segmented cells were manually excluded.

To compute the total fluorescence of a cell, the z-stack slice with the highest total intensity for that cell was selected. If the selected slice was either the first or the last one in the z-series, it was discarded. The total intensity of a cell was the computed by summing the pixel intensities inside the area determined by S.

To further remove outliers from the data, cells with extremely high intensity (the top 2.5% of cells ranked by intensity) were not considered. Cells having an area smaller than 0.5 times or larger than 1.5 times the median cell area were also removed as they are considered to be either too small or too large to be normal cells.

**Figure 2:**
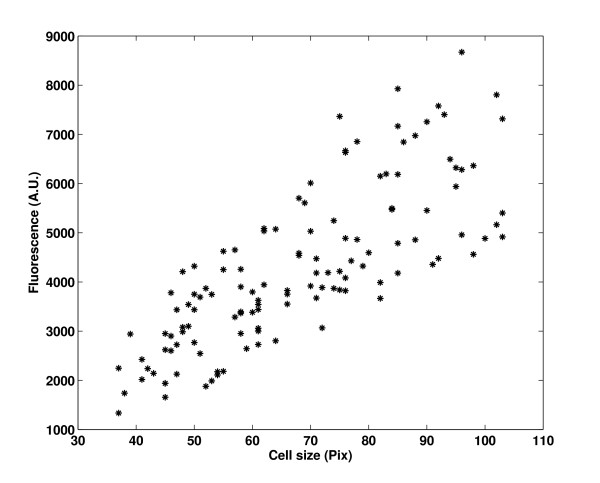
**Cellular background versus cell size**. Measured background fluorescence (cellular autofluorescence) versus cell size. This quantity can be assumed to depend linearly on the cell size when subtracting background.

After removing the outliers, we subtracted the background autofluorescence from the fluorescence levels of the cells. The background intensity is estimated by measuring the autofluorescence of λRED cells without the GFP insertion and then determining the mean background dependence on cell size. Figure 2 shows a typical measure of cellular autofluorescence and cell size from multiple individual cells.

Image analysis of cells with spots of RNA bound with MS2-GFP molecules requires additional steps. Segmentation of the cells is semi-automatic. First, each image was thresholded by the mean intensity. For the resulting binary mask, morphological opening with a disk-shaped structuring element was applied to remove noise pixels and the effect of image noise on the cell boundaries. Falsely segmented, e.g. clumped, cells, were manually excluded from the results. A spot detection algorithm based on kernel density estimates [29], was used to enhance the spots, which were segmented by the Otsu's method [30]. The number of RNA molecules in each spot is quantified by the spot intensity distribution slicing approach [19].

### Modelling gene expression with the delayed stochastic modelling strategy

Several steps in gene expression, such as transcript assembly, are time consuming [11]. Namely, the time scale of these processes is of comparable order of magnitude of an *E. coli *cell's lifetime. Also, some of the processes such as the assembly of the promoter open complex or protein folding and activations are multi-stepped complex processes that involve many reactions and events that cannot be accurately modelled as uni- or bimolecular reaction events.

However, from the point of view of the dynamics of RNA and protein production, they can be modelled as single-step delayed events [31]. For instance, between the binding of the RNA polymerase to the transcription start site and initiation of transcription elongation there is the process of promoter open complex formation [11]. Using the delayed stochastic simulation algorithm (delayed SSA), it is possible to model these processes as reaction events where the products are only completed a time interval after the reaction has initiated, instead of assuming them to be instantaneous bimolecular events [31,32]. This "delay" in the release of the products into the system can either be constant, i.e. the same for each of these reaction events, or be randomly drawn from a distribution each time the reaction occurs.

This delayed stochastic modelling strategy of gene expression and gene regulatory networks (GRNs) [31] accounts both for the stochasticity of the chemical interactions, as well as for the time length of events such as transcription and translation elongation, and it was shown to match gene expression dynamics at the single RNA and protein molecule level [7,33].

The delayed stochastic modelling strategy of GRNs can be implemented in the simulator SGNSim [34], and its dynamics is driven by the delayed SSA. Unlike the original SSA [35], this algorithm uses a waiting list to store delayed output events, proceeding as follows:

1) Set t = 0, t_stop _= stop time, read initial number of molecules and reactions, create empty waiting list L.

2) Do an SSA step for input events to get next reacting event R_1 _and corresponding occurrence time t_1_.

3) If t_1 _+ t < t_min _(the least time in L), set t = t + t_1_. Update number of molecules by performing R_1_, adding delayed products into L as necessary.

4) If t_1 _+ t > t_min_, set t = t_min_. Update number of molecules by releasing the first element in L.

5) If t < t_stop_, go to step 2.

Two assumptions are made by this modelling strategy. Since it is based on the original SSA, one is that the system of chemical reactions is well-stirred [35], and the other is that, once transcription is initiated, it is not aborted (this rate of abortions is likely below 1% in normal conditions) [31,36].

In our system, the promoter is tightly repressed by TetR dimer [10,37]. Induction is achieved by adding anhydrotetracycline (aTc) to the cell. When aTc binds to TetR, it forms the complex aTc-TetR. If this complex binds to the promoter, repression still occurs and the binding affinity is identical to that of TetR alone. However, the dissociation rate of aTc-TetR is much higher than the dissociation rate of TetR alone [38]. Consequently, the addition of aTc indirectly induces gene expression.

This system can be modelled in the delayed stochastic modelling strategy by the following set of reactions. Transcription and translation are modelled, respectively, by reactions 1 and 2 [33]. Reaction 3 is responsible for degradation of RNA molecules and reaction 4 is responsible for degradation of proteins. These reactions are assumed to be of the first order (the rate depends on the concentration of only one reactant), which was found to be a good approximation [33]. Note that, when a product X has a delay τ, represented by X(τ), it implies that when the reaction occurs, it takes τ seconds after that for X to be produced and become present in the cell:(1)(2)(3)(4)

In reactions 1-4, Pro is the promoter, RBS a ribosome binding site region of the RNA and P is a GFP molecule (thus directly correlated to the fluorescence observed).

The value of the rate constant k_1 _accounts for the number of available RNA polymerases in a cell, which is assumed to not vary during the measurements and thus is not explicitly represented. This rate is tuned empirically so as to match the mean expression levels at each concentration of inducers for which cells' expression levels were measured. Specifically, k_1 _was set to (in s^-1^): 1.5 × 10^-4^, 4.3 × 10^-4^, 1.4 × 10^-3^, 6.5 × 10^-3 ^and 2.8 × 10^-2 ^corresponding to the following concentrations of aTc (ng/ml): 0, 0.1, 0.5, 1 and 2, respectively.

Rate k_2 _is fixed at 0.19 s^-1^. This value accounts for the number of available ribosomes in *E. coli *under normal conditions and that in these bacteria, on average, the ratio between RBS to protein numbers is 1:1000 [8]. Rates k_3 _and k_4_, are the rates of degradation of RNA and proteins and are set to 0.004 s^-1 ^and 0.0002 s^-1^, respectively [22,23].

Two models were simulated. In the first model, all time delays are set to constant values, while in the second, the delay associated with the promoter open complex formation, τ_1_, is set to be a random variable following a Gamma distribution (with the mean value equal to the value of τ_1 _in model 1). The gamma distribution was used as it is the natural choice for modelling waiting times, given that the open complex process consists of a set of consecutive chemical reactions, each of which with an expected time to occur that are assumed to follow an exponential distribution [6]. The mean value of τ_1 _that best matched our measurements was found to be 19 s which is of the same order of magnitude of the value extracted from indirect measurements of its mean duration for P_LtetO-1 _[6].

All other delays are identical in the two models and were set to the following constant values: τ_2 _= 2 s and τ_3 _= 420 s [33]. These values allowed matching measurements of gene expression at the single protein level in *E. coli *[7] and account for the length of this gene (~760 nucleotides). Also, the maturation time of this protein is known to be less than 8 min and is accounted for in τ_3 _[39].

## Results

We measured the GFP levels of single cells for various concentrations of anhydrotetracycline (aTc). In Table 1 we inform on the mean and standard deviation of GFP levels of each cell population. Also shown are the relative mean GFP levels (normalized by the highest mean observed) and the Fano factor of GFP levels of individual cells in each condition. The Fano factor, defined as variance divided by the mean, is a common measure of diversity [40]. In all the cases, more than 50 cells were imaged.

**Table 1:** Summary of measurement results

aTc (ng/ml)	No. Cells	Mean GFP	STD GFP	Relative GFP	Fano Factor
0	245	1841.54	2414.88	0.01	3166.72

0.1	225	5526.12	3901.75	0.02	2754.85

0.5	88	16948.61	8781.03	0.07	4549.43

1	203	73338.24	33388.72	0.32	15200.89

2	299	231836.5	105391.2	1	47909.94

Summary of the experimental measurement results obtained using different inducer concentrations. Relative GFP is normalized by the highest mean level observed.

The Fano factor, while remaining approximately constant for weak induction strengths, increases for the two highest levels of induction. Note that it would not vary if transcription remained a Poissonian process for all levels of induction [41].

**Figure 3:**
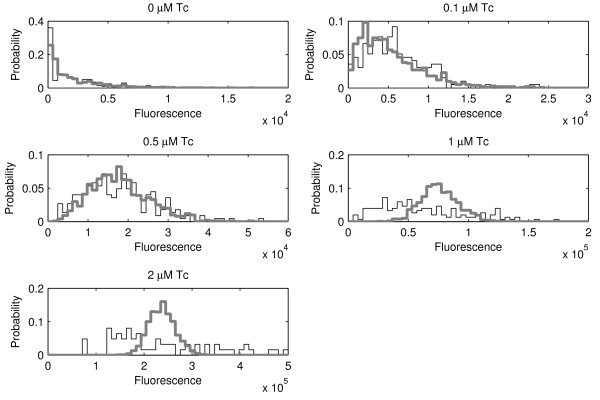
**Measured distributions compared with model distributions**. Binned distribution of the cells with given GFP expression levels for aTc (ng/ml) = 0, 0.1, 0.5, 1, and 2 (black lines). The probability is the fraction of cells in each bin. Also shown in each case is the distribution of expression levels as predicted by the model, imposing the same mean expression level as in the measurements (grey lines).

Given this observation, we next observed the distribution of GFP expression levels in the cells for each concentration of aTc (Figure 3) to better understand the source of diversity in gene expression levels. The expected distributions from the stochastic model with a constant time length for the promoter open complex formation are also shown for comparison. These are obtained by imposing the same mean expression levels as in the measurements.

Figure 3 shows that when setting the promoter delay to a constant value from one transcription event to the next, the model matches well the distributions of protein expression levels for the three lower rates of transcription induction. However, this is not true for the two higher rates. For these, the distributions of model and measurements do not match, in that the latter have much smaller variance.

Several possible causes can be ruled out for this discrepancy. First, increasing the amount of inducers does not affect the rate of translation events (or its elongation process). Due to this, the difference in the distributions is due to some event in transcription, either during transcription initiation or during transcription elongation, not accounted for in model 1.

The values of the rate of transcription initiation that allow the stochastic model to match the mean expression levels for all induction strengths in the measurements range from 1.4 × 10^-4 ^to 0.028 s^-1^. This implies that, in the model, consecutive transcription events are, for the strongest induction, separated, on average, by 35 seconds. This time interval is sufficiently long to assume that two RNA polymerases only rarely will collide on the DNA template [17,36]. The only tangible mechanism by which they could collide often would be the presence of a sequence-dependent pause (such as a *his *pause sequence) that would cause long transcriptional pauses to some, but not all, polymerases [42]. Such type of pause has not been reported to exist in the sequence coding for GFP used in our study. Thus, we rule out the occurrence of traffic events and bursts in transcription as a cause for the difference between the distributions of model 1 and measurements.

The mean duration of the open complex formation of P_LtetO-1 _was measured *in vitro *to be approximately 60 s [6]. While measures *in vitro *and *in vivo *may differ to some extent (likely, the process is more efficient *in vivo *than *in vitro*), it is safe to assume that, *in vivo*, the mean duration will be of the same order of magnitude as *in vitro*. Given this, it is reasonable to assume that the open complex is, for the higher rates of induction, a limiting step of transcripts production.

For the same reasons, we hypothesize that the variability of the duration of the promoter open complex formation, from one event to the next, may be the cause for the unexpected increase in Fano factor with induction strength. The range of variability of the duration of the open complex is currently unknown since only mean values have been measured, using *in vitro *experiments [11,16]. If the duration of this process has high variance for this particular promoter, then one would expect that it will introduce noise in gene expression [17], and thus contribute to cell-to-cell variability in protein levels.

**Figure 4:**
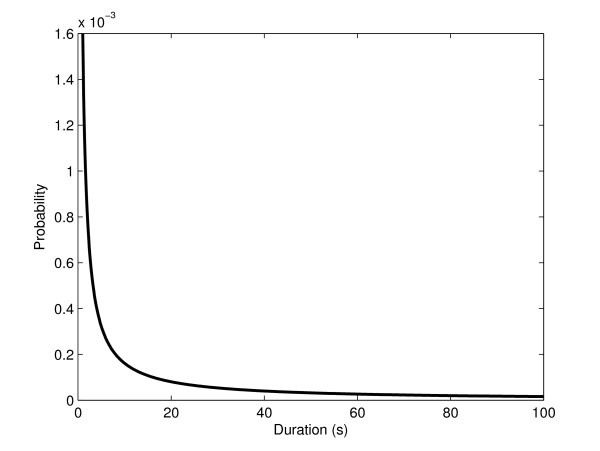
**Duration of promoter open complex formation**. Distribution of the values of the duration of the promoter open complex formation (τ_1_) in model 2.

**Figure 5:**
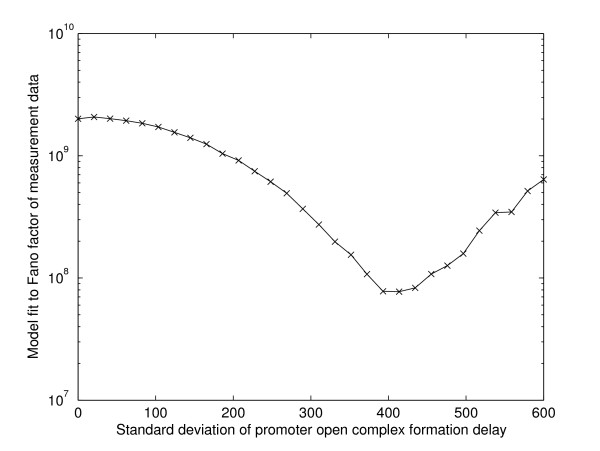
**Fit of model 2 to the measurements**. Distance between the Fano factors of model 2 and measurements as a function of the standard deviation of the duration of the promoter open complex formation (τ_1_).

We therefore hypothesize that the open complex formation has high variance in duration and test if a stochastic model where such variability is accounted for can match the measurements. Namely, to test if the variability in the duration of the open complex formation explains the observations, we simulated "model 2", in which the promoter delay is a random variable following a gamma distribution with mean of 19 s and a standard deviation of 400, giving the very fat-tailed distribution (found to best fit the observations) (Figure 4). In Figure 5 we show how well the model fits the measurements for different values of standard deviation of the duration of the promoter open complex formation. The fit *D *is calculated as the squared difference between the Fano factors of the measurements, *Fano(E)*, and the model, *Fano(M)*, summed over the five inductions strengths:(5)

**Figure 6:**
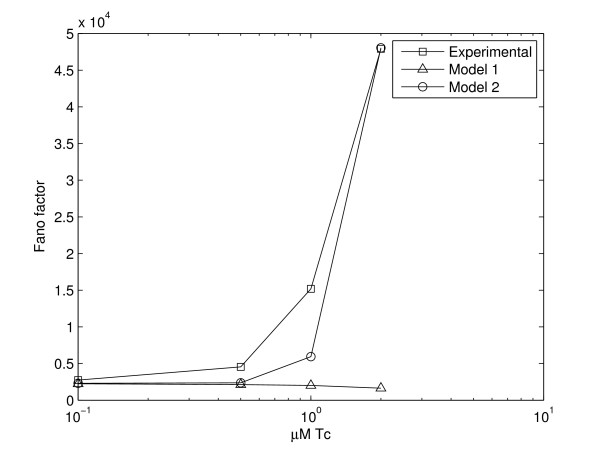
**Fano factors in experiments and models**. Fano factors for increasing induction strength in models and measurements.

Note, from Figure 4, that one expects many consecutive transcription events to be separated by very short time intervals, while few transcription events will be separated by very long time intervals. Mean expression levels do not differ between models 1 and 2, since they have the same mean promoter open complex duration and same rate of transcription initiation. In Figure 6 we plot the Fano factor from the measurements, from the model with constant promoter delay (model 1) and, from the model with a varying delay (model 2).

As seen in Figure 6, by accounting for the variability in the duration of the promoter open complex formation from one event to the next, model 2 accurately matches the distributions of protein expression observed in measurements for the entire range of values of induction strength. This allows concluding that the observed phenomenon may be due to non negligible effect of the promoter open complex formation in the dynamics of production of transcripts.

**Figure 7:**
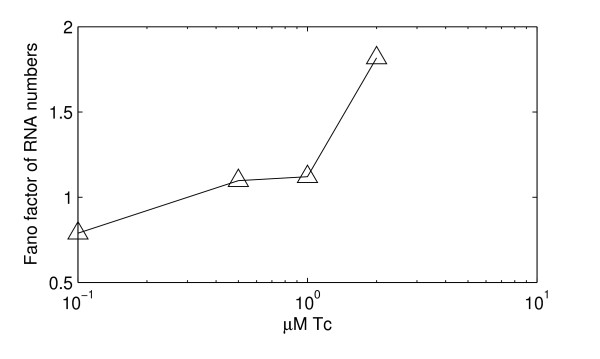
**Fano factors in measurements of RNA numbers**. Fano factors in RNA numbers for increasing induction strength in measurements.

If the variability of the promoter open complex step is responsible for the increase in Fano factor of GFP intensities in individual cells as induction strength is increased, then its effects ought to be visible also in the distribution of RNA numbers of the cell population. To verify this, we measured the transcriptional activity of a tetracycline inducible promoter, P_tet_, at the single RNA molecule level as described in the methods section. This measurement was made for the same levels of induction used to study the expression levels of GFP. The Fano factor of RNA numbers in individual cells for these levels of induction is shown in Figure 7. For induction strengths 0, 0.1, 0.5, 1 and 2 ng/ml the number of cells analyzed was 128, 185, 83, 124 and 248, respectively.

Comparing Figures 6 and 7, a clear resemble is visible between how the Fano factors of RNA and protein levels change with induction strength, providing strong evidence that the increase observed in the Fano factor of protein levels is due to the variability in duration of an event in transcription between consecutive transcription events. Likely, from all of the above, this event is the open complex formation, as the two processes are regulated by the same repressor-inducer system.

## Conclusions & Discussion

We observed an increase in cell-to-cell diversity in protein numbers as we increased transcription induction in a gene integrated into *E. coli *genome driven by P_LtetO-1_, a tetracycline inducible promoter. This increase is not expected if the process of transcripts' production is Poissonian [7,17,40,43]. The observed distribution of expression levels in individual cells indicates that the production of RNAs is not a Poisson-like process in the regime of strong induction. Relevantly, in this regime, the interval between transcription initiation events and the expected mean duration of the open complex formation of this promoter are of the same order of magnitude. Previous studies suggest that, in this scenario, the open complex formation will either function as a 'noise filter' or as a 'noise amplifier' of RNA and protein temporal numbers, depending on the degree of variability of its duration from one event to the next [17]. In the measurements, cell-to-cell variability in protein levels increased with induction, which suggests high variability in the time length of this process.

Thereafter, we compared the dynamics of stochastic models of gene expression with the measurements. The comparisons suggest that the variability in the duration of the promoter open complex formation is the most likely source of noise in the dynamics of RNA production in the regime of strong induction, and is responsible for enhancing the observed cell-to-cell diversity in protein numbers in the regime of strong induction. In this regard, it is stressed that, to the best of our knowledge, there are no possible events or mechanisms occurring during transcription or translation elongation, except for externally induced arrests, that, under normal conditions, would be responsible for the observed diversity in time intervals between the production of consecutive RNA and proteins [44,45].

To verify by independent means that the source of diversity in protein numbers for strong induction is in the dynamics of transcription initiation, at the level of the promoter, we measured directly the transcriptional activity of a tetracycline inducible promoter, P_tet_. For that, we placed this promoter to control the expression of an RNA sequence target for 96 MS2-GFP proteins. The Fano factor of these RNA numbers in individual cells changed with induction strength in a very similar manner to the Fano factor of GFP levels.

Our results suggest that the open complex formation of tetracycline inducible promoters is a process whose duration is highly variable from one event to the next and, therefore, is a non-negligible source of cell-to-cell variability in RNA and protein numbers in the regime of strong induction. Further studies are needed to determine if the dynamics of this mechanism is the only underlying cause for our observations. For example, we cannot completely rule out that our observations are due to some currently unreported increase in frequency of stochastic events in transcription elongation, such as sequence dependent pauses followed by premature terminations, due to the higher traffic of RNA polymerases in the strain. Note, this is not likely to be the case as the RNA coding for GFP and the RNA coding for MS2-GFP binding sites differ significantly, and the sequences prone for RNAp long-pausing are likely to be rare. Further, such sequences are not known to exist in the RNA coding for GFP used here.

We can also rule out other causes, such as overall cell-to-cell phenotypic diversity as a cause, as this would likely act at all induction strengths tested. Further, we can rule out "measurement noise" as a cause, as this would affect more strongly the regime of weak induction.

Most relevantly, so far most modelling strategies of gene expression for both prokaryote and eukaryote cells, assume gene expression to be an instantaneous process, and do not account for the duration of the various steps in transcription and translation [44], especially the promoter open complex formation, whose duration is likely a limiting factor of the number of transcription events in a given time interval. Our results suggest that both the noise and the cell-to-cell diversity in RNA and protein numbers are affected in a non negligible fashion by the dynamics of the promoter open complex formation.

## Authors' contributions

O-PS, HM, EL, SH, MK, MsK, SK, OY-H and ASR carried out the experiments, modelling and data analysis. ASR and O-PS conceived the study and wrote the manuscript. All authors read and approved the final manuscript.
